# The relevance of cultural diversity on safety culture: a CIRS data analysis to identify problem areas and competency requirements of professionals in healthcare institutions

**DOI:** 10.3205/zma001307

**Published:** 2020-03-16

**Authors:** Birgit Babitsch, Lisa Bretz, Hilke Mansholt, Nina-Alexandra Götz

**Affiliations:** 1 Osnabrück University, Department of New Public Health, Osnabrück, Germany

**Keywords:** cultural diversity, heterogeneous teams, competency requirements, patient safety, safety culture

## Abstract

**Aim: **The society and consequently also the health system have become increasingly culturally diverse which applies to both – patients and professionals. Studies indicate an influence of the ethnicity on the care context. With regard to this, considerably more knowledge is available regarding diversity among patients than among professionals, for example considering the effect of cultural diversity in teams. The impact of cultural diversity on patient safety has barely been investigated which means that potential effects as well as required measures and competencies cannot currently be specified. As part of the project “Gestaltungskompetenz als Innovator für hochzuverlässige Organisationen im Gesundheitssystem” (abbreviation: GIO, “Gestaltungskompetenz as an Innovator for High Reliability Organisations in the Healthcare System”) these questions are investigated in the context of a case study. Therefore, a CIRS data analysis was carried out to identify specific problem areas to derive competencies for the safe handling of cultural diversity between employees. On this basis and adjusted to the target group, an interactive learning management system will be developed for the advanced training of professionals.

**Method: **For the topic of cultural diversity, an analysis of the patient safety cases reported in CIRSmedical (Critical Incident Reporting System) was carried out followed by a qualitative summarising content analysis. The systematic search in CIRS was based on previously defined search terms as well as inclusion and exclusion criteria.

**Results:** 45 cases were included in the analysis. The results can be classed into two categories: “(Unsuccessful) communication” and “Unsuccessful adaptation to patient needs”. Cultural diversity was not usually named as a primary or explicit cause but usually a combination of several factors was given as the reason for the occurrence of an undesirable event.

**Conclusion: **The analysis of CIRS data identified concrete challenges resulting from the intercultural composition of teams and in the care context. The approaches for improvement should include both, organisational and personnel measures. In case of the latter, it is essential that competency requirements are identified resulting in suited offers for the competence development in the course of initial and professional development training for medical and nursing staff in ethnically diverse teams.

## Zusammenfassung

**Zielsetzung: **Die Gesellschaft und folglich auch das Gesundheitssystem sind zunehmend kulturell divers, was sich sowohl auf Seiten der Patient*innen als auch auf Seiten der Fachkräfte zeigt. Studien weisen auf einen Einfluss der Ethnizität im Versorgungskontext hin. Dabei liegen deutlich mehr Erkenntnisse für Patient*innen als für Fachkräfte, bspw. zum Einfluss kultureller Diversität in Teams, vor. Kaum untersucht ist der Einfluss kultureller Vielfalt auf die Patientensicherheit, so dass mögliche Effekte und notwendige Maßnahmen sowie erforderliche Kompetenzen derzeit nicht konkretisiert werden können. Im Rahmen des Projektes „Gestaltungskompetenz als Innovator für hochzuverlässige Organisationen im Gesundheitssystem“ (abgekürzt GIO) werden diese Fragen im Rahmen einer Fallstudie aufgegriffen. Hierzu wurde u. a. eine CIRS-Datenanalyse durchgeführt, um spezifische Problembereiche zu erkennen und darauf aufbauend Kompetenzen für einen sicheren Umgang mit kultureller Vielfalt zwischen Mitarbeitenden abzuleiten. Auf dieser Basis wird ein zielgruppengerechtes interaktives Lernangebot für die Weiterbildung entwickelt.

**Methodik: **Für den Bereich kulturelle Vielfalt wurde eine Analyse der in CIRSmedical (Critical Incident Reporting System) gemeldeten Patientensicherheitsfälle durchgeführt und zusammenfassend inhaltsanalytisch ausgewertet. Hierzu wurde eine systematische Suche mit vorab definierten Suchbegriffen und Einschluss- bzw. Ausschlusskriterien durchgeführt.

**Ergebnisse:** In die Auswertung wurden 45 Fälle eingeschlossen. Die Ergebnisse lassen sich zwei Kategorien zuordnen: „(Fehl-) Kommunikation“ und „Fehlerhafte Anpassung an Patientenbedürfnisse“. Kulturelle Vielfalt wurde meist nicht als primär ursächlicher oder expliziter Grund angegeben, sondern ein Zusammenspiel verschiedener Faktoren als Ursache für das Eintreten eines unerwünschten Ereignisses genannt.

**Schlussfolgerung: **Mit der Auswertung der CIRS-Daten lassen sich konkrete Herausforderungen, die sich durch die Interkulturalität in Teams und in Versorgungskontexten ergeben, benennen. Verbesserungsansätze sollten dabei sowohl organisationale als auch personale Maßnahmen beinhalten. Bei Letztgenannten ist es entscheidend, Kompetenzbedarfe zu identifizieren und gezielte Angebote zur Kompetenzentwicklung für die medizinische und pflegerische Aus- und Weiterbildung zu entwickeln für Mitarbeitende in ethisch diversen Teams.

## Introduction

The relationship between migration and health is multi-complex. It includes both, the experience and the occurrence of complaints and illnesses, as well as the perception and the utilisation of the health system [1], [2]. Ethnicity influences both, the patients as well as the professionals and due to the growth of cultural diversity it becomes high priority [3]. Culturally sensitive communication is particularly important in this context [4]. Existing concepts such as “(inter-)cultural competency” or “intercultural communication” mostly focus on the patients but rarely on the professionals [5], [6]. Additionally, in the context of patient safety, communication is of key importance [7], [8]. 

However, there is barely any information available on the influence of cultural diversity on patient safety or the safety culture in health facilities [9], [10]. It therefore remains uncertain, whether specific competencies are required for a patient safe handling of ethnically diverse patients. Furthermore, little is known about which influence cultural diversity has on the patient safety (positive or negative) and whether, or which culturally sensitive competencies employees working in heterogeneous teams require to ensure a patient safe working environment. For this purpose, the CIRS data analysis was carried out following the research question: “Which problem areas are reported in CIRSmedical in the context of cultural diversity?”

## Project description

In the context of the joint project “Gestaltungskompetenz als Innovator für hochzuverlässige Organisationen im Gesundheitssystem (GIO)” (=“Gestaltungskompetenz as an Innovator for High Reliability Organisations in the Healthcare System”) the influence of cultural diversity on the safety culture is investigated in a case study. The overreaching aim of the case study is to identify competencies for the safe handling of cultural diversity among employees and to use the results as the basis for the development of an interactive training offer. 

For this purpose, an analysis of patient safety cases reported in CIRSmedical (Critical Incident Reporting System) was carried out with regard to the topic “Ethnicity” [11]. For the systematic search the following search terms were used: “Migra*”, “Migri*”, “Herkunft” [“origin”], “Ethni*”, “Ausland” [“foreign”] or “Ausländ*” [“foreign”].

The CIRSmedical database comprised a total of 5,786 cases (as of 11th Jan 2018), with 190 cases identified by means of the used key words. After 145 cases had been excluded, 45 cases were included in the summarising qualitative content analysis according to Mayring [12]. The cases were independently evaluated by two scientists. Disagreements with regard to the inductively developed categories during the qualitative content analysis were resolved by consensus. 

## Results

The analysis of CIRS data showed, that problem areas linked to cultural diversity are often reported as causal for the undesirable event (next to others such as organisational factors). In 13 case descriptions, cultural diversity is named as the main factor for the issue and in ten cases it is named as a contributing factor. In 23 cases, cultural diversity is mentioned as a factor that is assumed to be contributing. For instance, next to a wrong medication, also language barriers contributed to an undesired event. 

**Figure 1:**
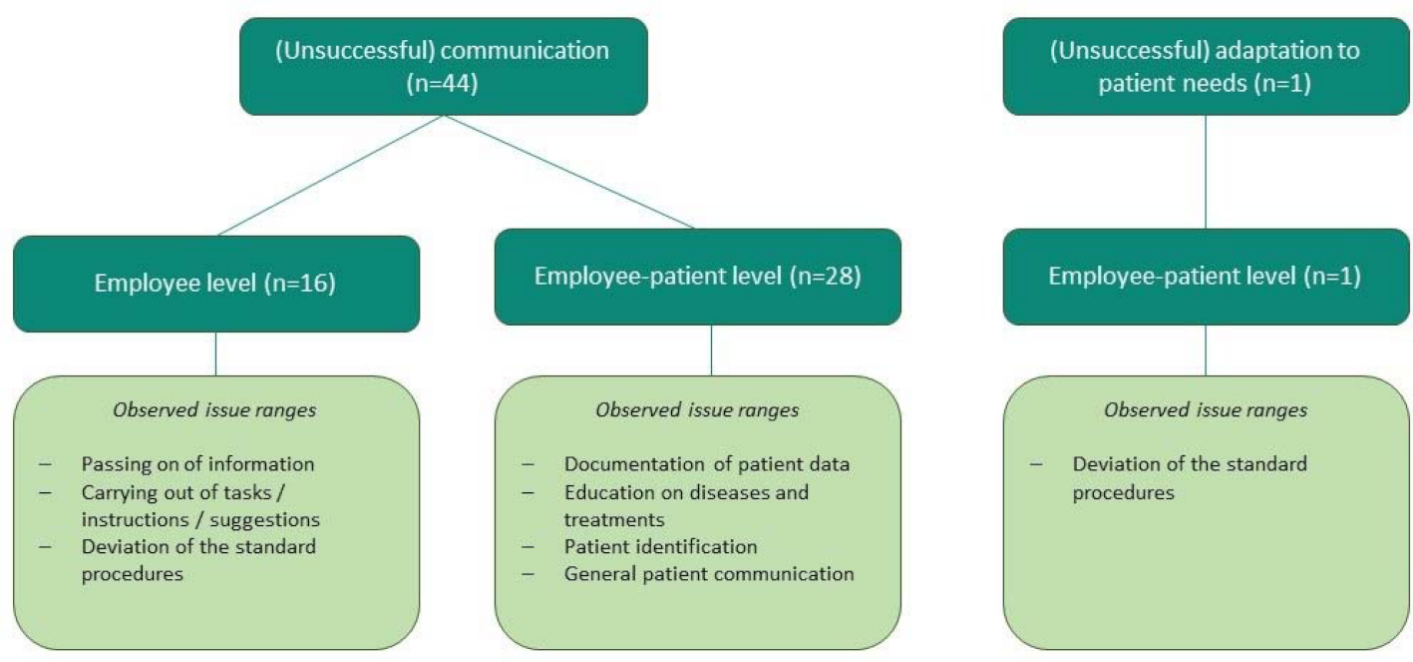
Summary of the CIRS results

The 45 identified cases were divided into two categories: “(Unsuccessful) communication” and “Unsuccessful adaptation to patient needs” (see figure 1 (Fig. 1)). 

In the latter category, a deviation from the standard procedure was made on account of patient needs. The patient safety was put at risk on account of respect for a patient's religion, which inhibited a marking of the limb to be operated. The category of “(Unsuccessful) communication” has been further subdivided in “Employee-employee communication” (1) and “Employee-patient communication” (2). The former includes cases in which information was insufficiently passed on, e.g. on account of language or labelling deficits. The latter includes cases which become apparent on account of language barriers such as general patient communication, the admission of patients and patient identification.

## Discussion

**Figure 2:**
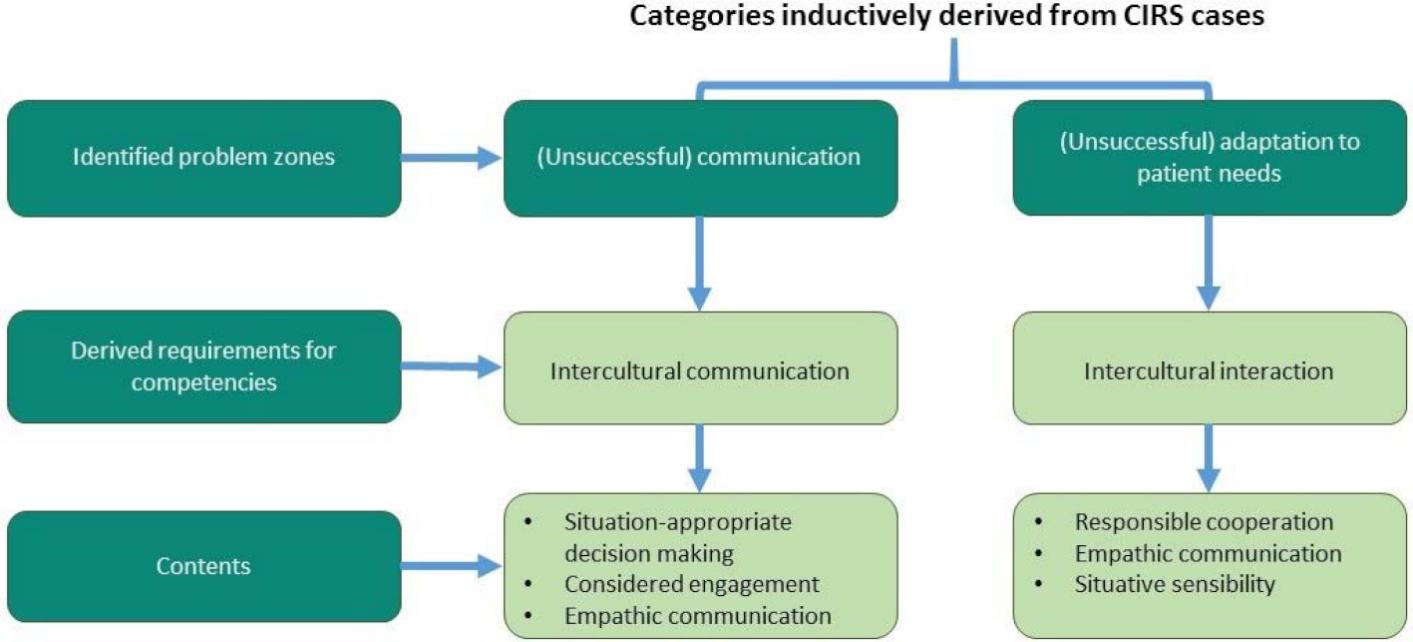
Requirements for competencies derived from CIRS cases

The results of the CIRS data analysis show that, due to communication problems in culturally diverse teams as well as difficulties caused by a lack of intercultural interaction, processes and therefore also patient safety can be put at risk. The approaches to improvement should include both, organisational and personnel measures. In case of the latter, it is essential that competency requirements are identified and suited offers for the competence advancement are developed for medical and nursing professional development training. In the following, the GIO project uses the inductively derived classifications to develop competency requirements and corresponding learning targets (see figure 2 (Fig. 2)), which in the next step will become the basis for the development of training offers in form of an interactive online learning environment. 

This is carried out on the basis of a competency catalogue developed in the GIO project. This competency catalogue is the first approach to specify competencies for the development of a safety culture, in the sense of a high reliability organisation. 

Limitations of the study are illustrated by the data protection and anonymisation of the cases in the CIRS database, edited by authorised employees of ÄZQ (Ärztliches Zentrum für Qualität in der Medizin=Agency for Quality in Medicine) in order to ensure, for instance, a description free of discrimination. Therefore, it is possible that additional problem areas remain undiscovered. However, the CIRS data analysis allows the assumption that culture and especially the communication on employee level are closely connected with and highly relevant for patient safety. 

## Conclusion

The CIRS data indicates that heterogeneity in healthcare and in particular communication represent competency requirements, for both, culturally heterogeneous teams as well as in the care of culturally diverse patients. However, these competencies have so far hardly been investigated. This also applies to the training of competencies in the context of the medical and nursing professional development training. Correspondingly, the combination of intercultural communication or intercultural interaction and safety culture for competence development might be an interesting topic to overcome the challenges faced by heterogeneous teams in the healthcare sector. 

## Funding

Funded by “Niedersächsisches Vorab”.

## Competing interests

The authors declare that they have no competing interests. 

## References

[1] Tagay S (2015). Andere Länder, andere Sitten: Patienten mit Migrationshintergrund verstehen. Dtsch Med Wochenschr.

[2] Spiess R, Kilcher A (2003). Arzt-Patienten-Interaktion im Migrationskontext. Praxis.

[3] Bouncken RB, Pfannstiel MA, Reuschl AJ, Haupt A (2015). Diversität managen. Wie Krankenhäuser das Beste aus personeller Vielfalt machen.

[4] Gesellschaft für Qualitätsmanagement in der Versorgung (2019). Sprachkompetenz in der Pflege.

[5] Walter C, Matar Z (2018). Interkulturelle Kommunikation in der Gesundheitswirtschaft. Herausforderungen, Chancen und Fallbeispiele.

[6] Roth J (2014). Interkulturelle Kompetenz in Gesundheit und Pflege.

[7] Aktionsbündnis Patientensicherheit (2006). Wege zur Patientensicherheit. Lernzielkatalog für Kompetenzen in der Patientensicherheit.

[8] Neudeck C (2019). Bremen: Forschungsprojekt Jacobs University Bremen: Patientensicherheit durch bessere Kommunikation.

[9] Milliken FJ, Martins LL (1996). Searching for Common Threads. Understanding the Multiple Effects of Diversity in Organizational Groups. Acad Manage.

[10] Die Beauftragte der Bundesregierung für Migration, Flüchtlinge und Integration (2015). Das kultursensible Krankenhaus. Ansätze zur interkulturellen Öffnung - PRAXISRATGEBER.

[11] CIRSmedical (2018). Ärztliches Zentrum für Qualität in der Medizin (ÄZQ).

[12] Mayring P (2015). Qualitative Inhaltsanalyse: Grundlagen und Techniken.

